# Identification of a novel disease-associated variant in the BRCA1 3’UTR that introduces a functional miR-103 target site

**DOI:** 10.1186/1897-4287-10-S2-A88

**Published:** 2012-04-12

**Authors:** BL Brewster, F Rossiello, JD French, SL Edwards, EM Wong, P Whiley, N Waddell, X Chen, B Bove, AB Spurdle, P Radice, AK Godwin, MC Southey, MA Brown, P Peterlongo

**Affiliations:** 1 School of Chemistry and Molecular Biosciences, The University of Queensland, Brisbane, Australia; 2 IFOM, Fondazione Istituto FIRC di Oncologia Molecolare, Via Adamello 16, 20139 Milan, Italy; 3 Department of Pathology, The University of Melbourne, Melbourne, Australia; 4 Queensland Institute of Medical Research, Brisbane, Australia; 5Fox Chase Cancer Center, Philadelphia, PA, USA; 5 Department of Pathology and Laboratory Medicine, University of Kansas Medical Center, Kansas City, KS, USA

## 

Mutations in the breast cancer susceptibility genes, *BRCA1* and *BRCA2*, represent the majority of the known familial breast cancer risk, yet account for only 20% of the total risk. As *BRCA1* is a large gene, genetic screening of high-risk individuals is limited to the coding regions and intron-exon boundaries, which precludes the identification of mutations in non-coding and untranslated (UTR) regions. Although mutations within 3’UTRs have been identified in many genes and are known to influence cancer susceptibility through the disruption or creation of protein and microRNA binding regions, mutation analysis of the *BRCA1* 3’UTR to date has been very limited. In this study, we screened the *BRCA1* 3’UTR for potential regulatory mutations. Using a large cohort of 1,585 *BRCA*-mutation negative, breast cancer cases, we identified seventeen novel *BRCA1* 3’UTR variants, eight of which were identified in breast cancer cases and absent in a large panel of cancer-free controls. Four of these variants, c.*58C>T, c.*528G>C, c.*718A>G, and c.*1271T>C, significantly reduced 3’UTR associated regulatory activity, as measured by reporter assays using MDA-MB-231 breast cancer cells. In addition, three *BRCA1* 3’UTR variants, c.*718A>G, c.*800T>C and c.*1340_42TGTdel, were predicted to create new miRNA binding sites. Of these, c.*1340_42TGTdel showed a significant reduction (25%, p=0.0007) in luciferase activity when co-expressed with the predicted targeting miRNA, *miR-103* in MCF-7 cells. This is the most comprehensive set of *BRCA1* 3’UTR variants published to date and highlights the importance of cataloguing 3’UTR variants for functional analyses and cancer risk association.

